# Supplementary material for: Non-cooperative 4E-BP2 folding with exchange between eIF4E-binding and binding-incompatible states tunes cap-dependent translation inhibition
Source: Nat Commun. 2020 Jun 19;11:3146. doi: 10.1038/s41467-020-16783-8 (PMC7305185; doi:10.1038/s41467-020-16783-8)
Supplement: Supplementary file 1 — Supplementary Information [file 41467_2020_16783_MOESM1_ESM.pdf]

Supplementary Information

Dawson *et* al (2020) Non-cooperative 4E-BP2 folding with exchange between eIF4E-binding and binding-incompatible states tunes cap-dependent translation inhibition

Supplementary Methods

Construct and sample generation

Primers for site-specific mutagenesis were synthesized by ACGT Corp (Toronto, ON). Forward primers are indicated with "_F" and reverse primers with "_R". The remaining constructs using in this paper were synthesized by ACGT Corp or GenScript.

Phosphorylation site primers

S65A_F: GGATCGTCGTAACgcgCCGATGGCGCAAACC
S65A_R: GGTTTGCGCCATCGGcgcGTTACGACGATCC

T70A_F: CCGATGGCGCAAgcgCCGCCGTGCCACC
T70A_R: GGTGGCACGGCGGcgcTTGCGCCATCGG

S83A_F: CATTCCGGGTGTGACCgcgCCGGGTACCCTGATTG
S83A_R: CAATCAGGGTACCCGGcgcGGTCACACCCGGAATG

Primers for smFRET (H32C/S91C C35S/C73S) and PRE (H32C C35S/C73S) constructs

H32C_C35S_F: GCAGCTACCTTGcGACTATTctACCAC
H32C_C35S_R: GTGGTagAATAGTCgCAAGGTAGCTGC

C35S_F: CTGCCGCATGACTACaGCACCACCCCGGGTGG
C35S_R: CCACCCGGGGTGGTGCtGTAGTCATGCGGCAG

C73S_F: GCAAACCCCGCCGaGCCACCTGCCGAACATTCC
C73S_R: GGAATGTTCGGCAGGTGGCtCGGCGGGGTTTGC

C73S_T70A_F: GCTCAGGCGCCACCCaGCCATCTGCCCAATATCC
C73S_T70A_R: GGATATTGGGCAGATGGCtGGGTGGCGCCTGAGC

S91C_F: GCCCTGGCACCTTAATTGAAGACTGCAAAGTAGAAG
S91C_R: CTTCTACTTTGCAGTCTTCAATTAAGGTGCCAGGGC

Hairpin mutant primers (^{37}TPX label indicates new hairpin sequence)

^{37}TPGGA_F: GACTACTGCACCACCCCGGGTGGCgcgCTGTTCAGC
^{37}TPGGA_R: GCTGAACAGcgcGCCACCCGGGGTGGTGCAGTAGTC

^{37}TPGGR_F: GACTACTGCACCACCCCGGGTGGCcgtCTGTTCAGC
^{37}TPGGR_R: GCTGAACAGacgGCCACCCGGGGTGGTGCAGTAGTC

^{37}TPGLT_F: CATTCCGGGTGTGACCaccCCGGGTctgACCCTGATTG
^{37}TPGLT_R: CAATCAGGGTcagACCCGGggtGGTCACACCCGGAATG

^{37}TPGP_F: GACTACTGCACCACCCCGGGTccgACCCTGTTCAGC
^{37}TPGP_R: GCTGAACAGGGTcggACCCGGGGTGGTGCAGTAGTC

^{37}TPGW_F: GACTACTGCACCACCCCGGGTtggACCCTGTTCAGC
^{37}TPGW_R: GCTGAACAGGGTccaACCCGGGGTGGTGCAGTAGTC

Supplementary Figures

Supplementary Figure 1

a.

```
4E-BP2                            *          *                    *     *               *
Human (Q13542)                33 DYCTTPGGTLFSTTPGGTRIIYDRKFLLDRRNSPMAQTPPCHLPNIPGVTSPGTLIED 90
Mouse (P70445)                33 DYCTTPGGTLFSTTPGGTRIIYDRKFLLDRRNSPMAQTPPCHLPNIPGVTSPGALIED 90
Rat (Q497A9)                  33 DYCTTPGGTLFSTTPGGTRIIYDRKFLLDRRNSPMAQTPPCHLPNIPGVTSPGALMED 90
Beaver(A0A250Y9B9)            33 DYCTTPGGTLFSTTPGGTRIIYDRKFLLDRRNSPMAQTPPCHLPNIPGVTSPGTLIED 90
Pig (A0A286ZLA1)              33 DYCTTPGGTLFSTTPGGTRIIYDRKFLLDRRNSPMAQTPPCHLPNIPGVTSPGTLIED 90
Cat (A0A2I2UNS0)              33 DYCTTPGGTLFSTTPGGTRIIYDRKFLLDRRNSPMAQTPPCHLPNIPGVTSPGTLIED 90
Dog (E2RPA3)                  33 DYCTTPGGTLFSTTPGGTRIIYDRKFLLDRRNSPMAQTPPCHLPNIPGVTSPGTLIED 90

4E-BP3
Human (O60516)                19 CYSTTPGGTLYATTPGGTRIIYDRKFLLECKNSPIARTPPCCLPQIPGVTTPPTAPLS 76
Mouse (Q80VV3)                19 GYSTTPGGTLYATTPGGTRIIYDRKFLLECKNSPIARTPPCCLPQIPGVTTLPAVPPS 76
Rat (D4AEG8)                  19 GYSTTPGGTLYATTPGGTRIIYDRKFLLECKNSPIARTPPCCLPQIPGVTIPPTVPPF 76
Beaver (A0A250Y8S4)           19 CYSTTPGGTPYATTPGGTRIIYDRKFLLECKNSPIARTPPCCLPQIPGVTTPLTAPTP 76
Pig (A0A4X1SEI3)              19 CYSSTPGGTLYATTPGGTRIIYDRKFLLECKNSPIARTPPCCLPQIPGVTTLPTAPSS 76
Cat (A0A337SDV7)              19 CYSTTPGGTLYATTPGGTRIIYDRKFLLECKNSPIARTPPCCLPQIPGVTTPPTAPPS 76
Dog (J9P5N5)                  19 CYSTTPGGTLYATTPGGTRIIYDRKFLLECKNSPIARTPPCCLPQIPGVTTPPTAPPS 76

4E-BP1
Human (Q13541)                33 DYSTTPGGTLFSTTPGGTRIIYDRKFLMECRNSPVTKTPPRDLPTIPGVTSPSSDEPP 90
Mouse (Q60876)                32 DYSTTPGGTLFSTTPGGTRIIYDRKFLMECRNSPVAKTPPKDLPAIPGVTSPTSDEPP 89
Rat (Q62622)                  32 DYSTTPGGTLFSTTPGGTRIIYDRKFLMECRNSPVAKTPPKDLPTIPGVTSPTSDEPP 89
Beaver (A0A250Y9H3)           33 DYSTTPGGTLFSTTPGGTRIIYDRKFLMECRNSPVAKTPPRDLPTIPGVTSPVSDEPP 90
Pig (A0A4X1VV86)              33 DYSTTPGGTLFSTTPGGTRIIYDRKFLMECRNSPVTKTPPRDLPTIPGVTSPVGDEPP 90
Cat (M3WD87)                  33 DYSTTPGGTLFSTTPGGTRIIYDRKFLMECRNSPVTKTPPRDLPTIPGVTSPTSDEPP 90
Dog (F1PZE3)                  33 DYSTTPGGTLFSTTPGGTRIIYDRKFLMECRNSPVTKTPPRDLPTIPGVTSPASEEPP 90

Invertebrate 4E-BP
Fruit Fly (Q9XZ56)            33 VYSSTPGGTLYSTTPGGTKLIYERAFMKNLRGSPLSQTPPSNV---PSCLLRGTPRTP 87
Silk moth (Q1HQ08)            32 VYSSTPGGTLYSTTPGGTKLIYERAFMKSLRQSPISQTPPQCAL--PAALLKNPSSVP 87
Europeansofttick(A0A293MWE1)  32 DYSSTPGGTIFSTTPGGSRIIYDRGFLMQMRNSPVARTPPKNLPVIPGVTLPSNSPDA 89
Black-legged tick (B7PTK2)    32 DYSSTPGGTIFSTTPGGSRIIYDRSFLMQMRNSPVARTPPKNLPLIPGVTVSCSPES- 88
Common tick (V5IHV4)          32 DYSSTPGGTIFSTTPGGSRIIYDRSFLMQMRNSPVARTPPKNLPLIPGVTVSCSPES- 88
Bush tick (I7GSF0)            32 DYSSTPGGTIFSTTPGGSRIIYDRSFLMQMRNSPVARTPPKNLPVIPGVTLSSSPDAS 89
Zebra tick (L7M3D3)           32 DYSSTPGGTIFSTTPGGSRIIYDRSFLMQMRHSPVARTPPKNLPVIPGVTLSSSPDVC 89
H. excavatum (A0A131XLX8)     32 DYSSTPGGTIFSTTPGGSRIIYDRSFLMQMRHSPVARTPPKNLPVIPGVTLSSSPDVC 89
Brown ear tick (A0A131YTP4)   32 DYSSTPGGTIFSTTPGGSRIIYDRSFLMQMRHSPVARTPPKNLPVIPGVTLSSSPDVS 89
R. zambeziensis(A0A224YML4)   32 DYSSTPGGTIFSTTPGGSRIIYDRSFLMQMRHSPVARTPPKNLPVIPGVTLSSSPDVS 89
Lone star tick (A0A0C9RS46)   32 DYSSTPGGTIFSTTPGGSRIIYDRSFLMQMRHSPVARTPPKNLPVIPGVTLSCSPDVS 89
A. triste (A0A023GER4)        32 DYSSTPGGTIFSTTPGGSRIIYDRSFLMQMRHSPVARTPPKNLPVIPGVTLSCSPDVA 89
Gulf Coast tick (G3MLT7)      32 DYSSTPGGTIFSTTPGGSRIIYDRSFLMQMRHSPVARTPPKNLPVIPGVTLSCSPDVA 89
A. aureolatum (A0A1E1X077)    32 DYSSTPGGTIFSTTPGGSRIIYDRSFLMQMRHSPVARTPPKNLPVIPGVTLSCSPDVS 89
A. sculptum (A0A1E1XUU8)      32 DYSSTPGGTIFSTTPGGSRIIYDRSFLMQMRHSPVARTPPKNLPVIPGVTLSCSPDVS 89
                                 Hairpin  Hairpin                                Pseudo-site
```

b. c.

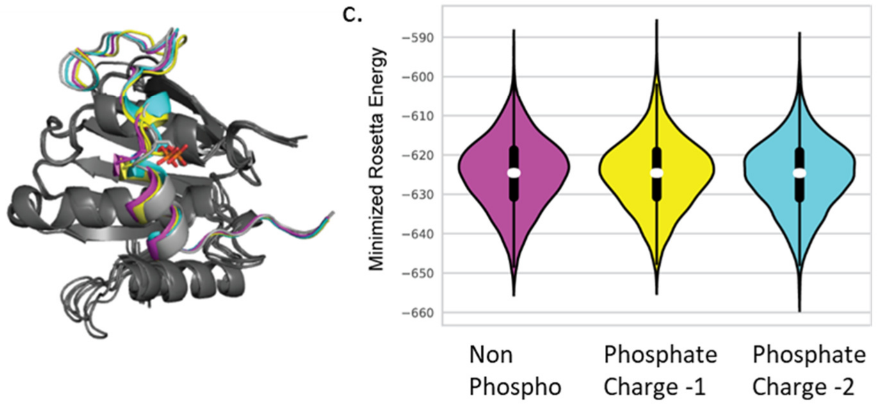

Supplementary Figure 1. Conservation of phospho sites and absence of electrostatic disruption of eIF4E:4E-BP by pS65 *in silico*. a. Conservation of phosphorylation sites, hairpin motifs and pseudo-site motifs in 4E-BP2, 4E-BP1, 4E-BP3, and invertebrate 4E-BPs. The 4E-BP1, 4E-BP2, and 4E-BP3 sequences were selected as examples from a consistent set of seven species: human, mouse, rat, beaver, pig, cat, and dog. The sequence UniProtKB identifiers are included with the its label. The two hairpins motifs (in red), which contain the T37 and T46 phosphorylation sites (using 4E-BP2 numbering), are conserved across 4E-BP 1, 2, and 3. The non-folding pseudo-site (in green) is similar to the hairpin motif, lacking a Gly in 4E-BP2. The pseudo-site is conserved in 4E-BP1 and 4E-BP2, containing phosphorylation site S83, but is less conserved for 4E-BP3. The invertebrate 4E-BP proteins, which have only one known isoform[1,2], lack the S83 phospho site. 4E-BP sequences from ticks have a TPGGS sequence instead of a TPGGT sequence at the second hairpin site. The other two phosphorylation sites (S65 and T70, in blue) are conserved across isoforms. The protein sequences are from the UniProt Database[3], found using the database's BLASTP server[4]. Sequence alignments were performed using CLUSTAL OMEGA[5]. b and c. Energy minimization of phospho and non-phospho 4E-BP bound to eIF4E. Rosetta score minimization against the energy function was used to gauge the potential for electrostatic repulsion in bound state structures of S65 phosphorylation. Five thousand minimized structures were generated for each state starting from atom coordinates in the human eIF4E:4E-BP1 structure (PDB ID 4UED). Two charge states for phosphate were attempted, at charge -1 and -2. Panel B shows an overlay of the best scoring structure from each set superimposed to PDB:4UED, with eIF4E in dark-gray for all sets, and 4E-BP colored in dark-gray for PDB:4UED, cyan for the best scoring non-phospho Rosetta model, magenta for phosphate charge -1, and yellow for phosphate charge -2, demonstrating that phosphorylation does not require perturbation of the overall structure. Panel C shows that the modeled energy distributions are statistically consistent between states, even with different net charges are used on the phosphate. The boxes in Supplementary Figure 1c

show the interquartile range of energy scores, with a white dot labeling the median. Source data for Supplementary Figure 1a are provided as a Source Data file.

Supplementary Figure 2

a. Close-up views of five-phospho 4E-BP2 acid denaturation at 20°C

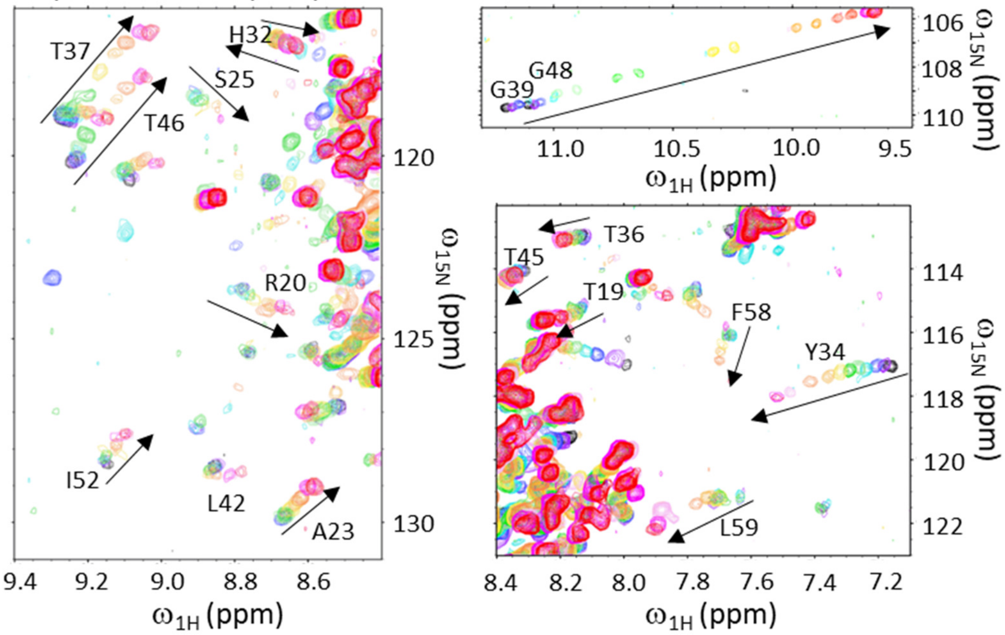

pH2.2 pH2.6 pH3.0 pH4.0 pH4.5 pH5.0 pH5.5 pH6.0 pH6.4 pH6.8 pH7.2 pH8.0

b. Thermal denaturation of non-phospho 4E-BP2 at pH5.0

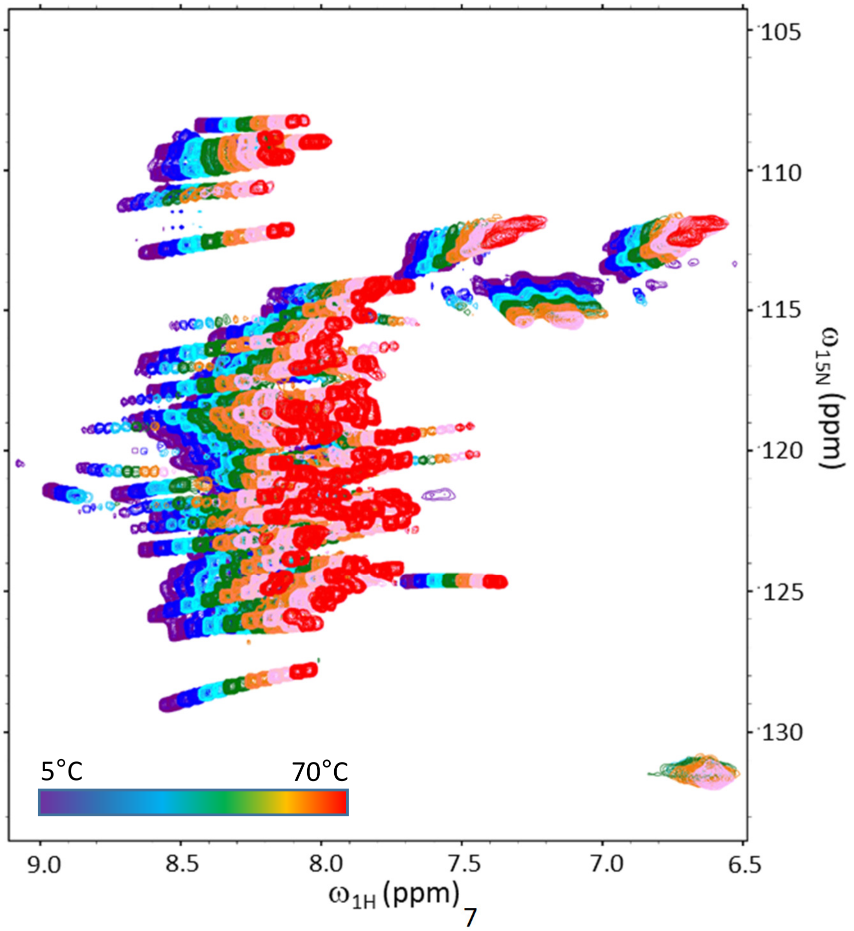

Supplementary Figure 2. Acid denaturation of five- and non-phospho 4E-BP2. a. Acid denaturation of the folded domain of five-phospho 4E-BP2 at 20°C. Chemical shift changes, $\Delta\omega$, due to pH are shown in overlaid NMR HSQC spectra. Peaks that are shifting toward random coil values (ω_{1H} ~8.2 ppm) are indicated with arrows and correspond to residue undergoing acid denaturation. b. Thermal denaturation of disordered non-phospho 4E-BP2 at pH5.0.

Supplementary Figure 3

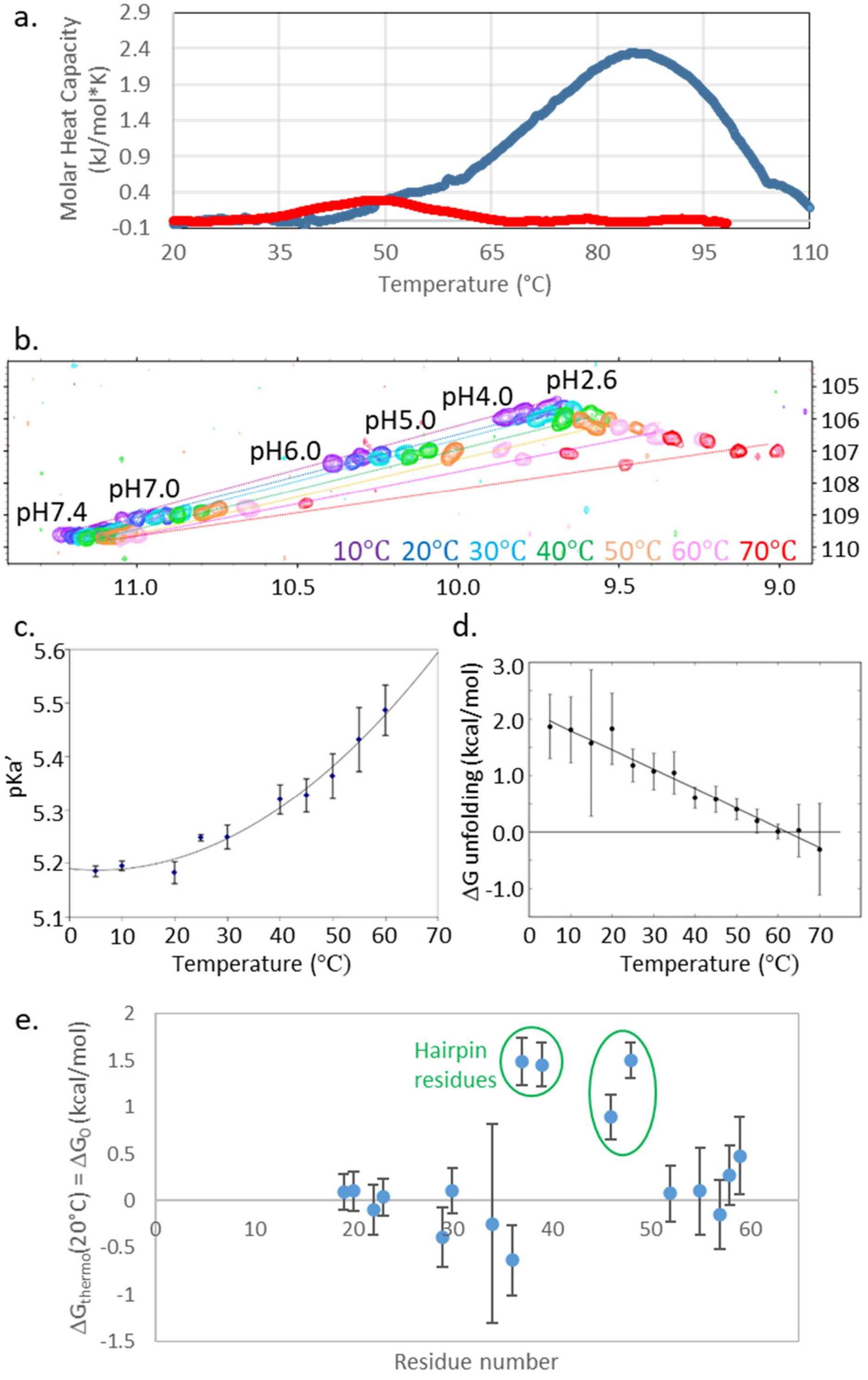

Supplementary Figure 3. DSC and NMR thermal denaturation analysis. a. DSC data for five-phospho 4E-BP2 at pH 7.4 (blue) and pH 1.0 (red). The data were buffer-corrected and baseline-corrected. b. Overlay of thermal denaturation NMR data for G39 and G48 (10°C to 70°C data shown) taken for samples at H 7.4, 7.0, 6.0, 5.0, 4.0, and 2.6. The acid denaturation titrations for G39 and G48 peaks are approximately marked by dashed guidelines with colors indicating specific temperatures. c-e. Demonstration of thermal denaturation fitting method on G39 (Methods). c. Temperature-dependent apparent pK_a values and error bars from fits of acid denaturation data at different temperatures. The plateau of apparent pK_a at low temperatures is used to estimate the true pK_a of acid denaturation. d. $\Delta G(T)$ for unfolding versus temperature, which are fitted to two-state thermal denaturation energies. The error bars are derived from pKa(T) errors via propagation of error e. Chart shows ΔG_{thermo} at 20°C, which corresponds to ΔG_0 in the thermal denaturation fitting analysis. Error bars are from propagation of error. Source data are provided as a Source Data file.

Supplementary Figure 4

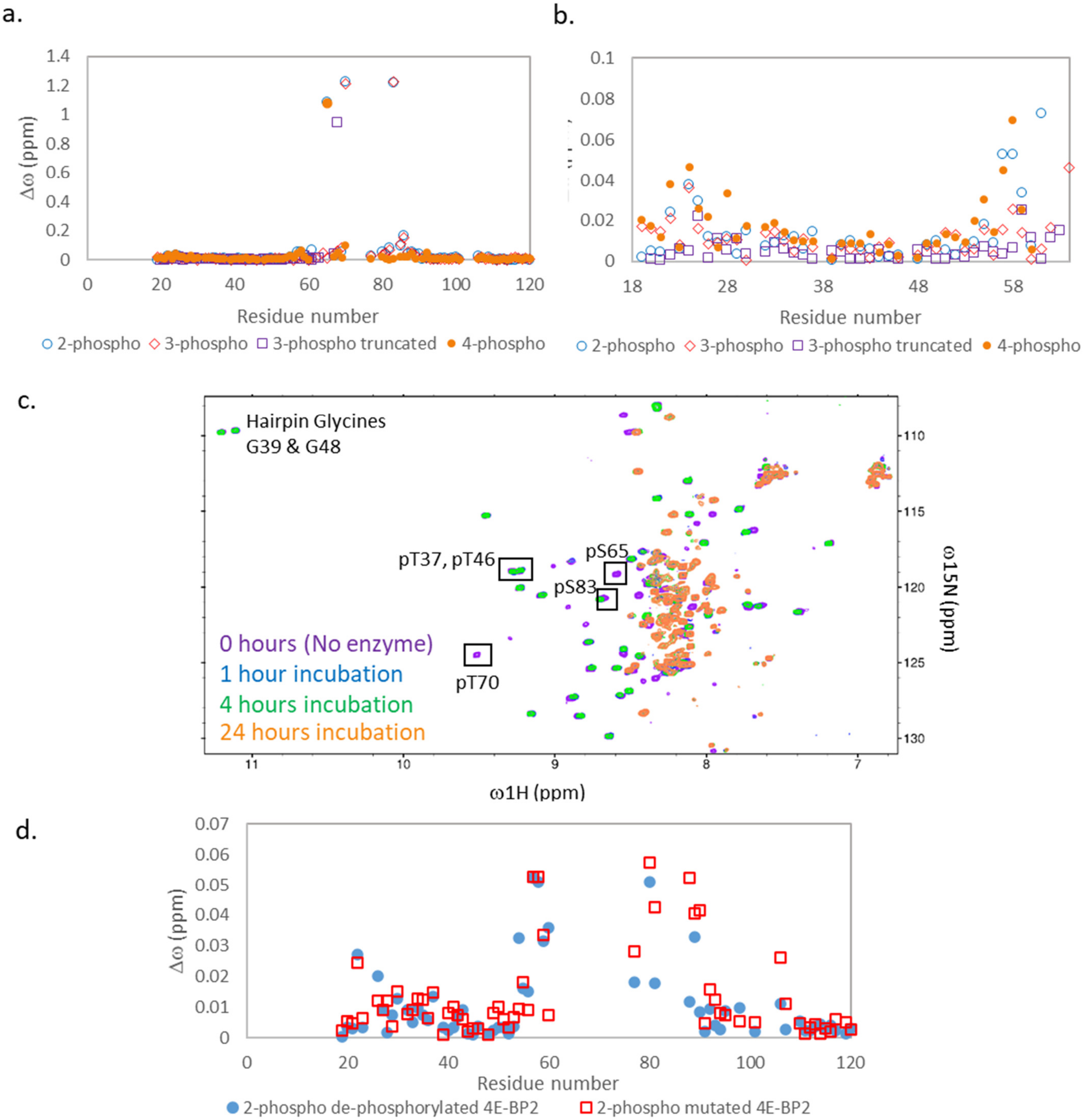

Supplementary Figure 4. Backbone (1H-15N) chemical shift changes due to C-IDR modifications. a. Chemical shift changes, $\Delta\omega$, for 4E-BP2 phosphorylation site mutants, referenced to five-phospho 4E-BP2 (See Equation (1), Methods). All constructs are full-length unless stated otherwise. Two-phospho 4E-BP2 is pT37/pT46. Three-phospho is pT37/pT46/pS65. Four phospho 4E-BP2 is pT37/pT46/pT70/pS83. Three-phospho truncated 4E-BP2 contains residues 1-67, phosphorylated at pT37/pT46/pS65. b. Enlargement of the Supplementary Figure 4a plot showing the smaller $\Delta\omega$'s within the folded domain, between residues 18-64, away from the mutation sites. c. Dephosphorylation of five-phospho p4E-BP2. Overlay of NMR ^{15}N-^{1}H HSQC spectra of five-phospho 4E-BP2 (pH 7.4, 25°C) at zero, one, four, and 24-hours incubation with the Lambda Protein phosphatase. pS65, pT70, and pS83 have already been de-phosphorylated after one hour. pT37, pT46, and the hairpin glycine resonances remain after four hours incubation. The folded domain peaks are also observed. Total de-phosphorylation is only observed in the long-term (24 hour) incubation sample. d. Comparison of $\Delta\omega$ values for enzymatically de-phosphorylated two-phospho 4E-BP2 (4 hour incubation, data shown as blue circles) and mutated (S65A/T70A/S83A) two-phospho mutated 4E-BP2 (open red squares), both referenced to the five-phospho WT 4E-BP2 spectrum. Source data are provided as a Source Data file.

Supplementary Figure 5

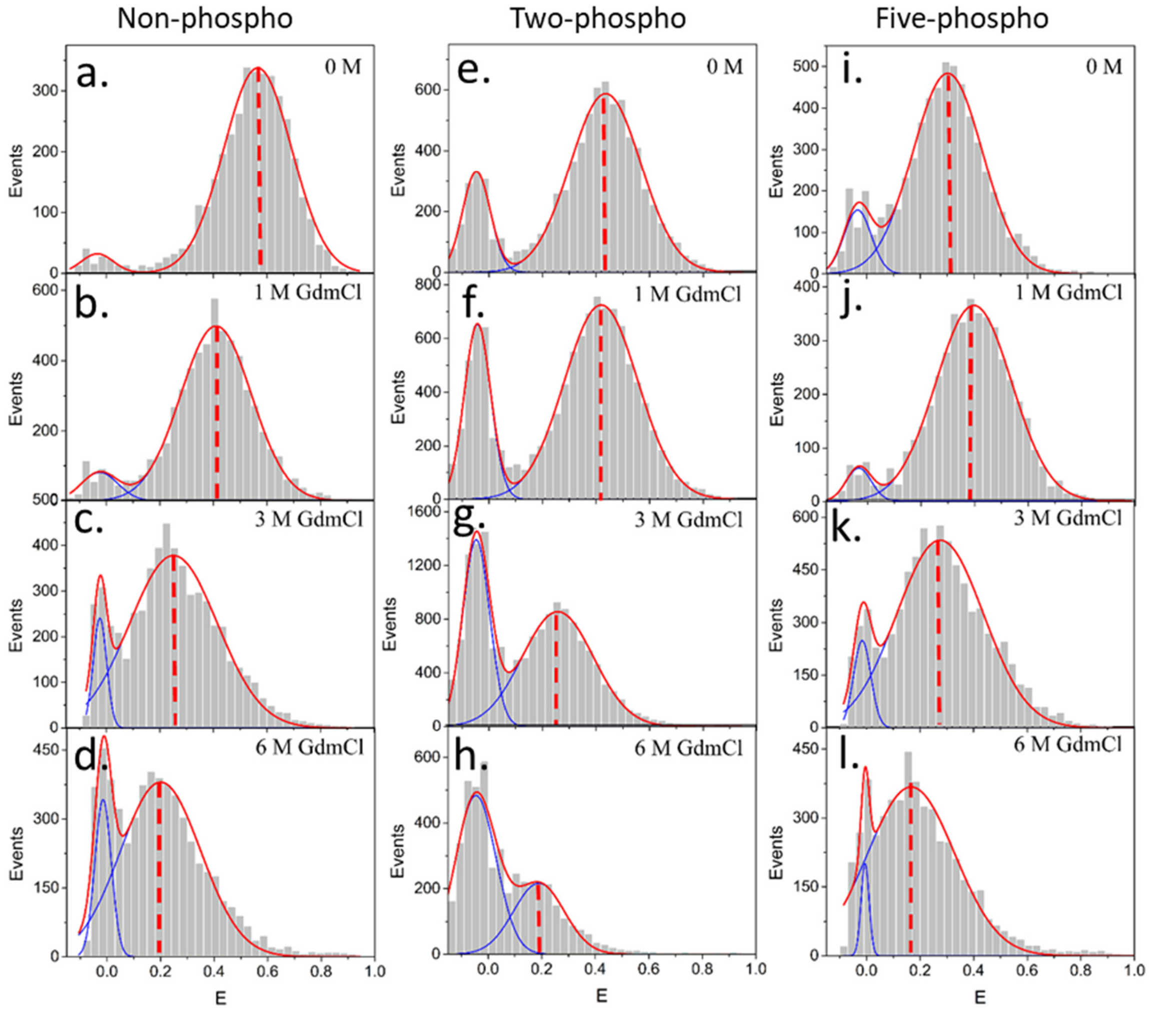

Supplementary Figure 5. smFRET data. smFRET efficiency histograms of non-phospho (a-d), two-phospho (e-h), and five-phospho (i-l) 4E-BP2 at different concentrations of GdmCl. Solid lines are Gaussian fits to the data; red solid lines are the overall fitting profiles and blue lines are individual Gaussians peaks. The minor peak near $E \approx 0$ corresponds to molecules without (active) acceptors. The red dash lines indicate the locations of the peak FRET efficiency for the non-zero Gaussian.

Supplementary Figure 6

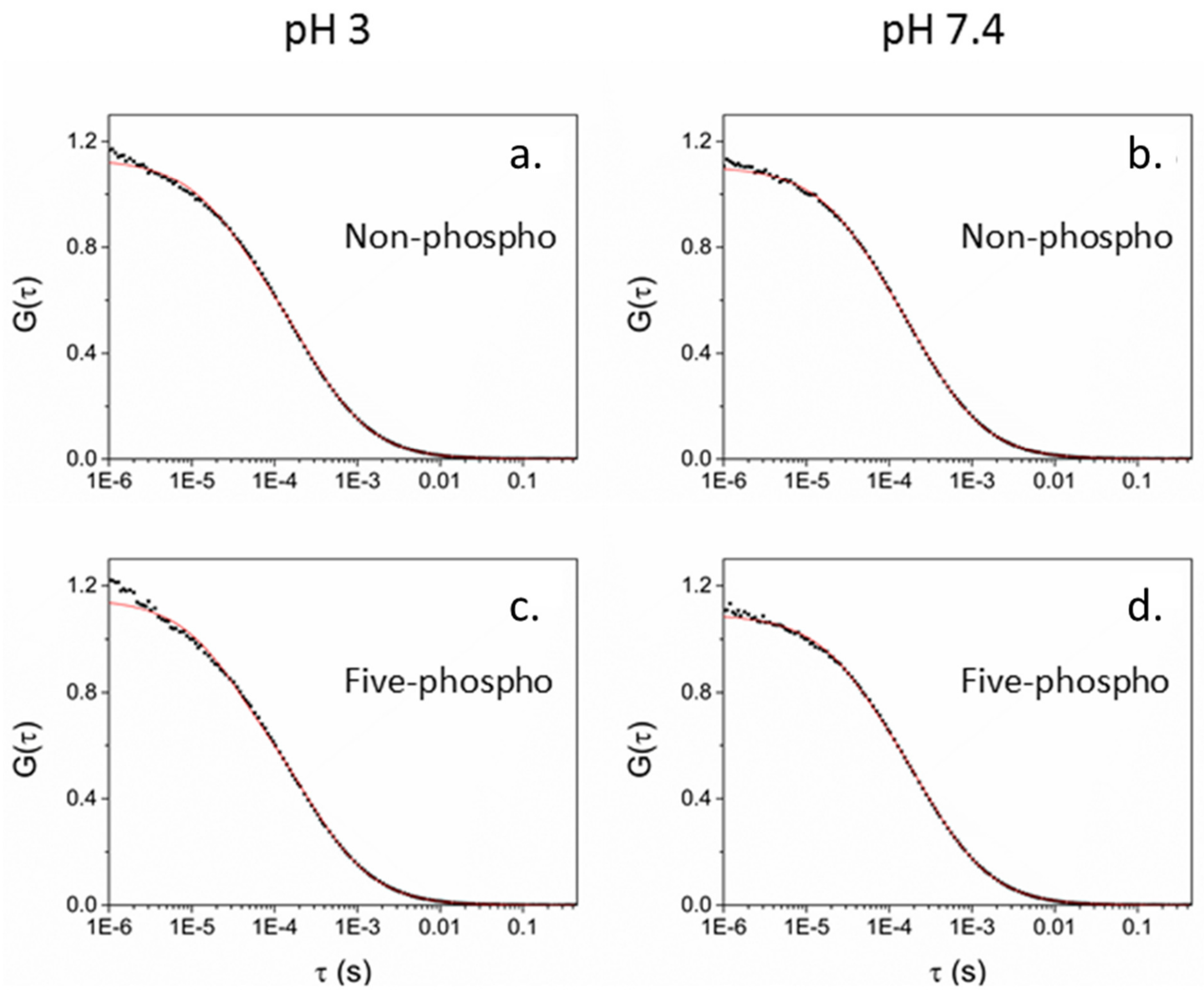

Supplementary Figure 6. Fluorescence Correlation Spectroscopy (FCS) data. FCS of non-phospho (a-b) and five-phospho (c-d) 4E-BP2 on the same dye-labelled samples used for smFRET measurements. Experimental data (black dots) were acquired at pH 3 and 7.4 upon the excitation of the donor at 488 nm and were fitted to the typical model of molecular diffusion and dye photophysics (red curves), as described previously[6]. Hydrodynamic radii (R_H) obtained from the fitting varied in a narrow range between 27.6 Å and 31.0 Å, indicating the absence of significant protein aggregation at low pH.

Supplementary Figure 7

Construct	K_D (nM)	Protein diagram	Reference
Non-phospho	3.2 ± 0.6	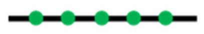	13
pT37	7.2 ± 1.7	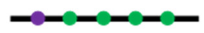	13
pT46	2.4 ± 0.5	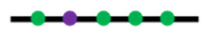	13
pS65 pT70 pS83 (T37A/T46A)	11.3 ± 2.9	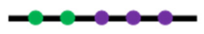	13
G39V/G48V Five-phospho	36.1 ± 3.5	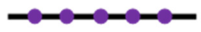	13
pT37 pT46 (Two-phospho)	267 ± 32	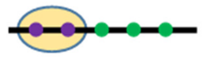	13
pT37 pT46 pS83 (S65A/T70A)	1800	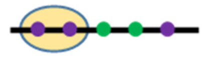	Current paper
pT37 pT46 pT70 (S65A/S83A)	2700 ± 500	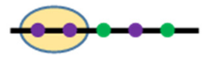	Current paper
pT37 pT46 pS65 (T70A/S83A)	4000 ± 2000	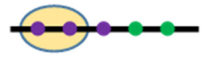	Current paper
pT37 pT46 pT70 pS83 (S65A)	1930 ± 200	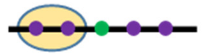	Current paper
pT37 pT46 pS65 pS83 (T70A)	23000 ± 12000	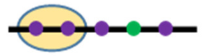	Current paper
pT37 pT46 pS65 pT70 (S83A)	5700 ± 1500	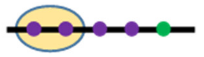	Current paper
Five-phospho	15000 ± 4000	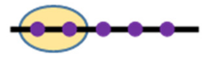	13, current paper

● :Phosphorylated Group ● :Non-phosphorylated group 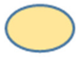 :Folded domain

Supplementary Figure 7. eIF4E:4E-BP2 binding affinities from ITC. eIF4E:4E-BP2 binding affinities for various 4E-BP2 mutations and phosphorylation states obtained from ITC binding studies. The K_D values were measured using ITC (see Methods). The third column shows schematic cartoons for each of the 4E-BP2 variants. Green circles indicate non-phosphorylated sites, purple circles are phosphorylated residues, and the tan oval represents the folded domain of 4E-BP2.

Supplementary Figure 8

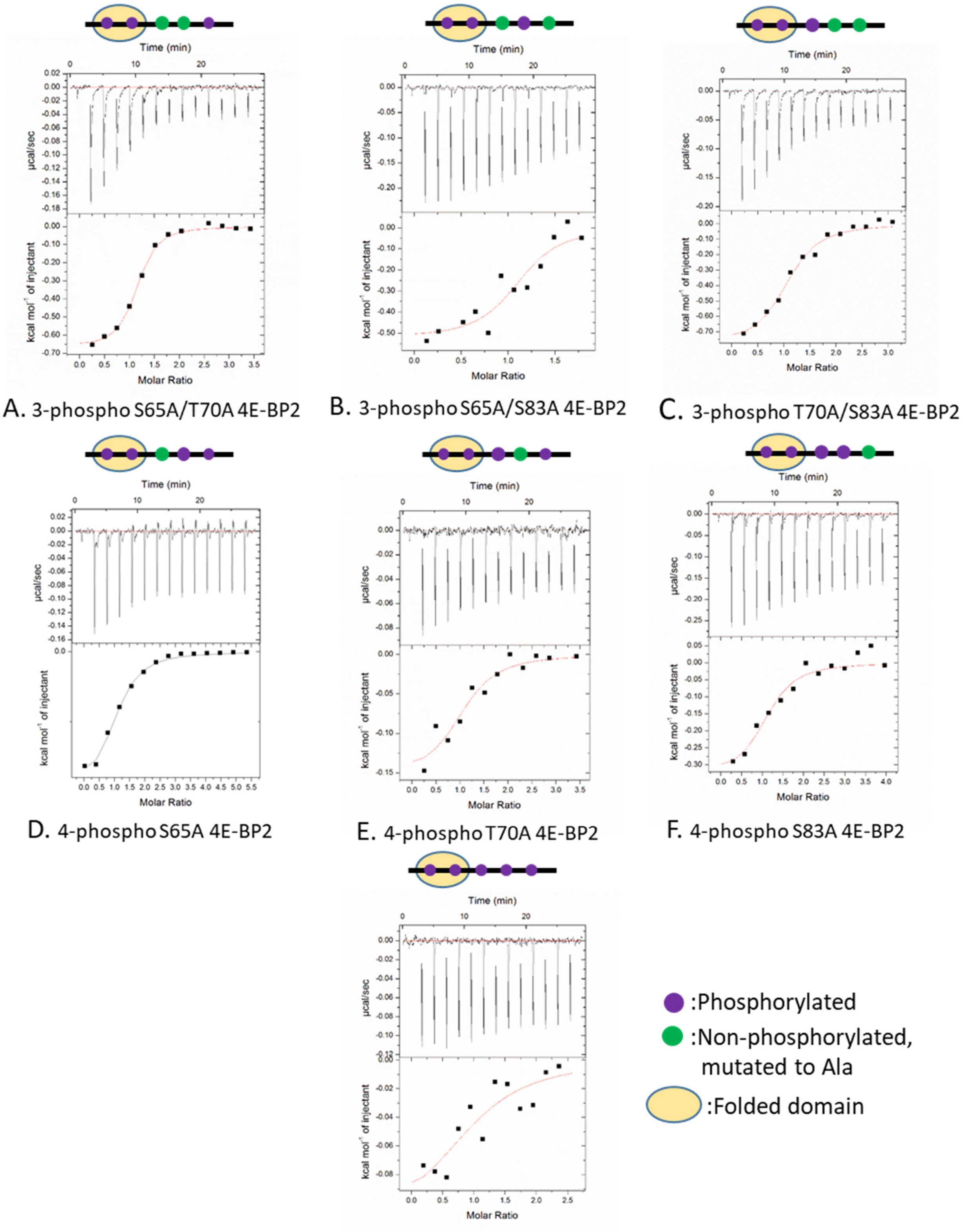

A. 3-phospho S65A/T70A 4E-BP2

B. 3-phospho S65A/S83A 4E-BP2

C. 3-phospho T70A/S83A 4E-BP2

D. 4-phospho S65A 4E-BP2

E. 4-phospho T70A 4E-BP2

F. 4-phospho S83A 4E-BP2

G. 5-phospho wildtype 4E-BP2

Supplementary Figure 8. ITC thermograms of eIF4E:4E-BP2 binding. ITC thermograms of eIF4E binding to various C-IDR phosphorylation states of 4E-BP2. The K_D values and their uncertainties were measured using ITC (see Methods) as the mean and standard deviation of sample repeats.

Supplementary Figure 9

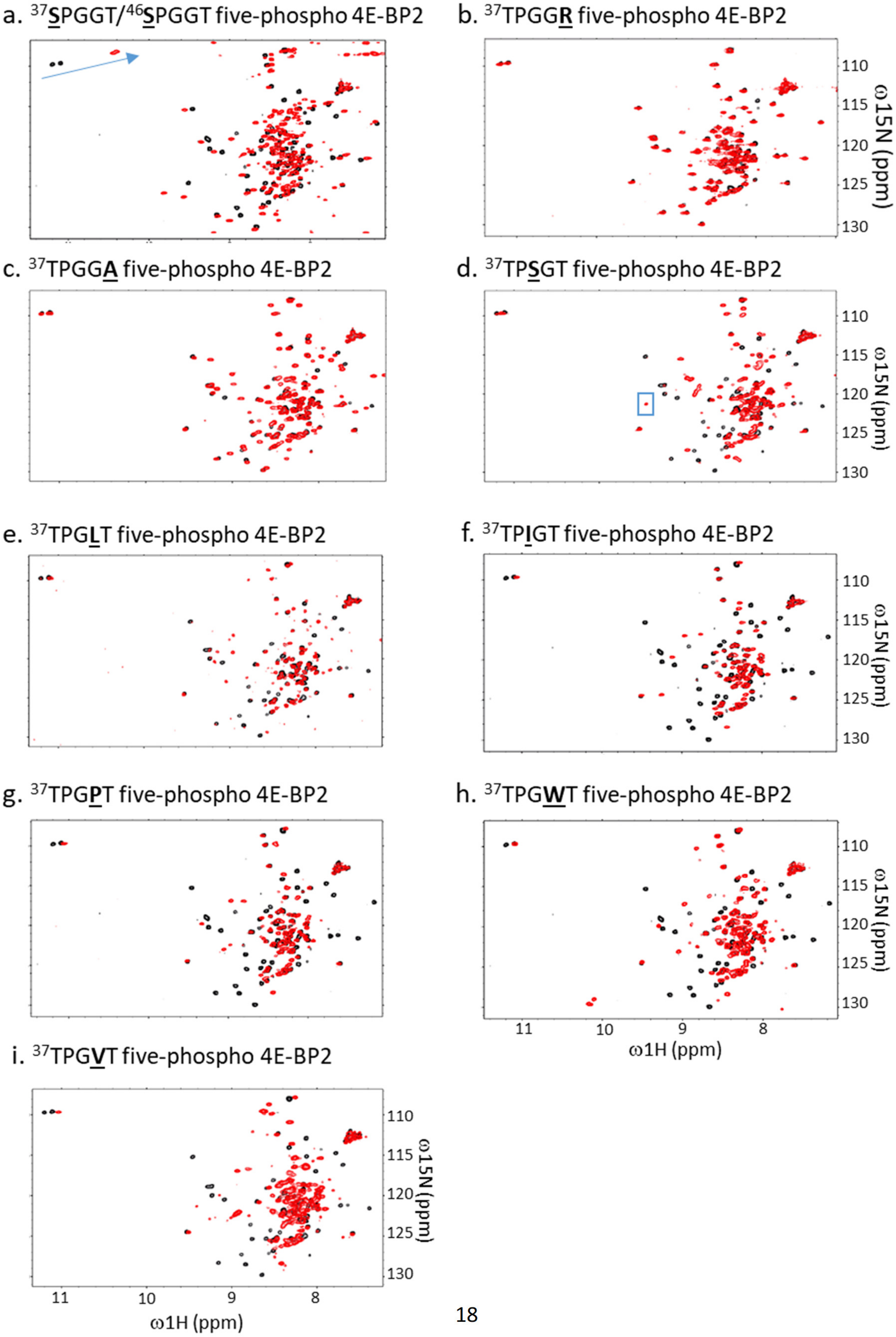

Supplementary Figure 9. Effects of mutation on hairpin ($^{37/46}$TPGGT) motifs in 4E-BP2. The number indicates the first residue of the motif. Spectra for each mutant (in red) are shown overlaid with a WT five-phospho 4E-BP2 spectrum (in black) taken under the same buffer and temperature conditions. a. ^{37}SPGGT/^{46}SPGGT five-phospho 4E-BP2 NMR spectrum showing the presence of the two characteristic $^{39/48}$Gly peaks, but they are not as down-field shifted in $^{37/46}$SPGGT as in $^{37/46}$TPGGT, suggesting that the latter is more stable. b. ^{37}TPGGR five-phospho 4E-BP2 NMR spectrum. c. ^{37}TPGGA five-phospho 4E-BP2 NMR spectrum. d. ^{37}TPSGT five-phospho 4E-BP2 NMR spectrum. The probable hairpin serine (G39S) is noted by a blue box. e. ^{37}TPGLT five-phospho 4E-BP2 NMR spectrum. f. ^{37}TPIGT five-phospho 4E-BP2. g. ^{37}TPGPT five-phospho 4E-BP2. h. ^{37}TPGWT five-phospho 4E-BP2. i. ^{37}TPGVT five-phospho 4E-BP2. The ^{37}SPGGT/^{46}SPGGT 4E-BP2 and matching WT 4E-BP2 spectra were taken at pH 6.0 and 20°C. The other spectra were at pH7.4 and 25°C.

Supplementary Table 1. pKa fits for acid denaturation data at 20°C

Residue	pKa1	pKa2	ω1 (ppm)	ω2 (ppm)	ω3 (ppm)	Chi sq	comment
T19	5.11 ± 0.07	-	25.898 ± 0.004	26.003 ± 0.003	-	0.001067	2 state
A23	4.2 ± 0.1	-	28.672 ± 0.004	28.467 ± 0.006	-	0.001291	2 state
A28	3.63 ± 0.02	-	27.318 ± 0.003	26.642 ± 0.003	-	0.001131	2 state
Q29	5.1 ± 0.1	*2.3 ± 0.4 **	25.455 ± 0.002	25.548 ± 0.007	25.44 ± 0.05	0.000567	3 state
H32	*6.0 ± 0.2 **	*2.6 ± 0.1 **	26.640 ± 0.008	26.713 ± 0.003	26.631 ± 0.006	0.000275	3 state
D33	*6.0 ± 0.1 **	-	26.045 ± 0.006	26.156 ± 0.002	-	0.001031	2 state
Y34	*6.1 ± 0.3 **	4.0 ± 0.1	25.223 ± 0.009	25.38 ± 0.02	25.702 ± 0.003	0.000945	3 state
C35	4.04 ± 0.07	-	27.645 ± 0.002	27.526 ± 0.008	-	0.000688	2 state
T36	5.4 ± 0.1	-	25.555 ± 0.003	25.612 ± 0.003	-	0.001407	2 state
T37	4.98 ± 0.07	-	27.596 ± 0.007	27.03 ± 0.01	-	0.009491	2 state
G39	5.17 ± 0.01	-	27.98 ± 0.01	25.932 ± 0.005	-	0.006196	2 state
L42	5.3 ± 0.1	-	128.49 ± 0.01	128.742 ± 0.007	-	0.005707	2 state, ω15N data
T45	5.1 ± 0.1	*3.3 ± 0.6 **	25.916 ± 0.004	26.05 ± 0.02	25.92 ± 0.02	0.0009	3 state
T46	4.98 ± 0.06	-	27.747 ± 0.008	27.23 ± 0.01	-	0.008335	2 state
G48	5.13 ± 0.01	-	28.093 ± 0.009	25.989 ± 0.008	-	0.007017	2 state
I52	4.2 ± 0.2	-	28.937 ± 0.008	28.758 ± 0.007	-	0.003558	2 state
R56	4.4 ± 0.2	-	125.94 ± 0.01	124.91 ± 0.02	-	0.067199	2 state, ω15N data
K57	4.87 ± 0.04	-	125.76 ± 0.01	124.79 ± 0.02	-	0.011677	2 state, ω15N data

Supplementary Table 1. Fitted pKa values for chemical shift changes of backbone amide groups during pH titration. Titrations associated with unfolding were identified by their peak shifts toward random coil at lower pH (Supplementary Figure 2a). Some residues were affected by additional protonation events (e.g. D33 being perturbed by H32's sidechain titration), whose pKa values are indicated with asterisks. Source data are provided as a Source Data file.

Supplementary Table 2. Standard free energies, entropies, and change in heat capacity for thermal denaturation

Residue	ΔG⁰ (kcal/mol)	error	ΔS⁰ (kcal/mol*K)	error	ΔCp (kcal/mol*K)	error	Chi sq
19	0.09	0.19	0.03	0.02	0.07	1.01	0.39
20	0.10	0.21	0.02	0.02	-0.41	0.44	0.25
22	-0.10	0.27	0.00	0.02	0.40	0.43	0.19
23	0.04	0.20	0.00	0.01	0.15	0.28	0.38
29	-0.39	0.32	-0.02	0.02	0.48	0.81	0.98
30	0.11	0.24	-0.01	0.03	0.32	0.42	0.34
34	-0.24	1.05	-0.01	0.08	-0.01	0.84	0.30
36	-0.63	0.38	-0.02	0.03	0.19	0.45	0.55
37	1.49	0.25	0.02	0.02	0.16	0.36	0.44
39	1.46	0.23	0.03	0.02	0.01	0.27	0.23
46	0.89	0.24	0.00	0.02	0.14	0.29	0.15
48	1.50	0.19	0.05	0.02	-0.12	0.32	0.03
52	0.08	0.30	-0.01	0.03	0.19	0.46	0.39
55	0.10	0.46	0.01	0.05	0.34	0.82	1.32
57	-0.15	0.37	0.01	0.04	-0.15	1.33	0.25

*Units are degrees Celsius, kcal/mol, and ppm for temperatures, free energies, and chemical shifts, respectively.

Supplementary Table 2. NMR thermal denaturation analysis fitting values.

Standard free energies, entropies, and change in heat capacity for thermal denaturation. In the second analysis step, ΔG(T) for unfolding was found by using pKa'(T), the true pKa value (estimated using the low temperature plateau in pKa'(T) values, see Methods), and Equation (10). See Supplementary Figure 3d for an example of ΔG(T) versus temperature data. ΔG(T) versus temperature was fitted to Equation (7) (a standard equation for the thermodynamics of protein unfolding) for ΔG^0, ΔS^0, and ΔCp at a reference temperature of T0=20°C. The extracted ΔG^0 values for residues in the folded domain are plotted as ΔG_{thermo}(T0=20°C) versus residue number in Supplementary Figure 3e. Source data are provided as a Source Data file.

Supplementary References

1. Kume, A. et al. RNAi of the translation inhibition gene 4E-BP identified from the hard tick, Haemaphysalis longicornis, affects lipid storage during the off-host starvation period of ticks. *Parasitol Res* **111**, 889-96 (2012).

2. Lasko, P. The drosophila melanogaster genome: translation factors and RNA binding proteins. *J Cell Biol* **150**, F51-6 (2000).

3. UniProt: a worldwide hub of protein knowledge. *Nucleic Acids Res* **47**, D506-D515 (2019).

4. Madden, T. The BLAST Sequence Analysis Tool. . in *The NCBI Handbook* (eds. McEntyre, J. & Ostell, J.) (National Center for Biotechnology Information (US), 2002).

5. Sievers, F. et al. Fast, scalable generation of high-quality protein multiple sequence alignments using Clustal Omega. *Mol Syst Biol* **7**, 539 (2011).

6. Zhang, Z. *Single-Molecule Spectroscopy of Disordered States and Dynamics in Proteins* (University of Toronto, Toronto, 2017).
