# Supplementary material for: Non-cooperative 4E-BP2 folding with exchange between eIF4E-binding and binding-incompatible states tunes cap-dependent translation inhibition
Source: Nat Commun. 2020 Jun 19;11:3146. doi: 10.1038/s41467-020-16783-8 (PMC7305185; doi:10.1038/s41467-020-16783-8)
Supplement: Supplementary file 3 — Description of Additional Supplementary Files [file 41467_2020_16783_MOESM3_ESM.docx]

**Description of Additional Supplementary Files**

**File Name: Supplementary Data 1**

**Description:** Apparent pKa’ fits for combined analysis of acid and thermal denaturation. In step one of the thermal denaturation analysis, apparent pKa values were fitted at multiple temperatures.
