# Supplementary material for: Non-cooperative 4E-BP2 folding with exchange between eIF4E-binding and binding-incompatible states tunes cap-dependent translation inhibition
Source: Nat Commun. 2020 Jun 19;11:3146. doi: 10.1038/s41467-020-16783-8 (PMC7305185; doi:10.1038/s41467-020-16783-8)
Supplement: Supplementary file 6 — Source Data [file 41467_2020_16783_MOESM6_ESM.zip › Source_data/4EBP_alignment.tar!/align/align_121519.docx]

```
4E-BP2				       *        *                  *    *            *
Human (Q13542)			33 DYCTTPGGTLFSTTPGGTRIIYDRKFLLDRRNSPMAQTPPCHLPNIPGVTSPGTLIED 90
Mouse (P70445)	   		33 DYCTTPGGTLFSTTPGGTRIIYDRKFLLDRRNSPMAQTPPCHLPNIPGVTSPGALIED 90
Rat (Q497A9)	   		33 DYCTTPGGTLFSTTPGGTRIIYDRKFLLDRRNSPMAQTPPCHLPNIPGVTSPGALMED 90
Beaver(A0A250Y9B9)  		33 DYCTTPGGTLFSTTPGGTRIIYDRKFLLDRRNSPMAQTPPCHLPNIPGVTSPGTLIED 90
Pig (A0A286ZLA1)	   		33 DYCTTPGGTLFSTTPGGTRIIYDRKFLLDRRNSPMAQTPPCHLPNIPGVTSPGTLIED 90
Cat (A0A2I2UNS0)	   		33 DYCTTPGGTLFSTTPGGTRIIYDRKFLLDRRNSPMAQTPPCHLPNIPGVTSPGTLIED 90
Dog (E2RPA3)	   		33 DYCTTPGGTLFSTTPGGTRIIYDRKFLLDRRNSPMAQTPPCHLPNIPGVTSPGTLIED 90

4E-BP3
Human (O60516)			19 CYSTTPGGTLYATTPGGTRIIYDRKFLLECKNSPIARTPPCCLPQIPGVTTPPTAPLS 76
Mouse (Q80VV3)	   		19 GYSTTPGGTLYATTPGGTRIIYDRKFLLECKNSPIARTPPCCLPQIPGVTTLPAVPPS 76
Rat (D4AEG8)	   		19 GYSTTPGGTLYATTPGGTRIIYDRKFLLECKNSPIARTPPCCLPQIPGVTIPPTVPPF 76
Beaver (A0A250Y8S4) 		19 CYSTTPGGTPYATTPGGTRIIYDRKFLLECKNSPIARTPPCCLPQIPGVTTPLTAPTP 76
Pig (A0A4X1SEI3)	   		19 CYSSTPGGTLYATTPGGTRIIYDRKFLLECKNSPIARTPPCCLPQIPGVTTLPTAPSS 76
Cat (A0A337SDV7)	   		19 CYSTTPGGTLYATTPGGTRIIYDRKFLLECKNSPIARTPPCCLPQIPGVTTPPTAPPS 76
Dog (J9P5N5)	   		19 CYSTTPGGTLYATTPGGTRIIYDRKFLLECKNSPIARTPPCCLPQIPGVTTPPTAPPS 76


4E-BP1
Human (Q13541)			33 DYSTTPGGTLFSTTPGGTRIIYDRKFLMECRNSPVTKTPPRDLPTIPGVTSPSSDEPP 90
Mouse (Q60876)	   		32 DYSTTPGGTLFSTTPGGTRIIYDRKFLMECRNSPVAKTPPKDLPAIPGVTSPTSDEPP 89
Rat (Q62622)	   		32 DYSTTPGGTLFSTTPGGTRIIYDRKFLMECRNSPVAKTPPKDLPTIPGVTSPTSDEPP 89
Beaver (A0A250Y9H3) 		33 DYSTTPGGTLFSTTPGGTRIIYDRKFLMECRNSPVAKTPPRDLPTIPGVTSPVSDEPP 90
Pig (A0A4X1VV86)	   		33 DYSTTPGGTLFSTTPGGTRIIYDRKFLMECRNSPVTKTPPRDLPTIPGVTSPVGDEPP 90
Cat (M3WD87)	   		33 DYSTTPGGTLFSTTPGGTRIIYDRKFLMECRNSPVTKTPPRDLPTIPGVTSPTSDEPP 90
Dog (F1PZE3)	   		33 DYSTTPGGTLFSTTPGGTRIIYDRKFLMECRNSPVTKTPPRDLPTIPGVTSPASEEPP 90

Tick 4E-BP
European soft tick (A0A293MWE1)	32 DYSSTPGGTIFSTTPGGSRIIYDRGFLMQMRNSPVARTPPKNLPVIPGVTLPSNSPDA 89
Black-legged tick (B7PTK2)	32 DYSSTPGGTIFSTTPGGSRIIYDRSFLMQMRNSPVARTPPKNLPLIPGVTVSCSPES- 88
Common tick (V5IHV4)		32 DYSSTPGGTIFSTTPGGSRIIYDRSFLMQMRNSPVARTPPKNLPLIPGVTVSCSPES- 88
Bush tick (I7GSF0)		32 DYSSTPGGTIFSTTPGGSRIIYDRSFLMQMRNSPVARTPPKNLPVIPGVTLSSSPDAS 89
Zebra tick (L7M3D3)		32 DYSSTPGGTIFSTTPGGSRIIYDRSFLMQMRHSPVARTPPKNLPVIPGVTLSSSPDVC 89
H. excavatum (A0A131XLX8)		32 DYSSTPGGTIFSTTPGGSRIIYDRSFLMQMRHSPVARTPPKNLPVIPGVTLSSSPDVC 89
Brown ear tick (A0A131YTP4)	32 DYSSTPGGTIFSTTPGGSRIIYDRSFLMQMRHSPVARTPPKNLPVIPGVTLSSSPDVS 89
R. zambeziensis(A0A224YML4)	32 DYSSTPGGTIFSTTPGGSRIIYDRSFLMQMRHSPVARTPPKNLPVIPGVTLSSSPDVS 89
Lone star tick (A0A0C9RS46)       	32 DYSSTPGGTIFSTTPGGSRIIYDRSFLMQMRHSPVARTPPKNLPVIPGVTLSCSPDVS 89
A. triste (A0A023GER4)          	32 DYSSTPGGTIFSTTPGGSRIIYDRSFLMQMRHSPVARTPPKNLPVIPGVTLSCSPDVA 89
Gulf Coast tick (G3MLT7)    	32 DYSSTPGGTIFSTTPGGSRIIYDRSFLMQMRHSPVARTPPKNLPVIPGVTLSCSPDVA 89
A. aureolatum (A0A1E1X077)  	32 DYSSTPGGTIFSTTPGGSRIIYDRSFLMQMRHSPVARTPPKNLPVIPGVTLSCSPDVS 89
A. sculptum (A0A1E1XUU8)    	32 DYSSTPGGTIFSTTPGGSRIIYDRSFLMQMRHSPVARTPPKNLPVIPGVTLSCSPDVS 89
				      Hairpin  Hairpin                            Pseudo-site
```
